# Understanding Transferable Supply Chain Lessons and Practices to a “High-Tech” Industry Using Guidelines from a Primary Sector Industry: A Case Study in the Food Industry Supply Chain

**DOI:** 10.1155/2015/198385

**Published:** 2015-03-03

**Authors:** Adrian E. Coronado Mondragon, Christian E. Coronado Mondragon, Etienne S. Coronado

**Affiliations:** ^1^School of Management, Royal Holloway, University of London, Egham TW20 0EX, UK; ^2^Marine Institute of Memorial University of Newfoundland, St. John's, NL, Canada A1C 5RE; ^3^Networking and Telecommunications Professional Services, Montreal, QC, Canada H3S 1T2

## Abstract

Flexibility and innovation at creating shapes, adapting processes, and modifying materials characterize composites materials, a “high-tech” industry. However, the absence of standard manufacturing processes and the selection of materials with defined properties hinder the configuration of the composites materials supply chain. An interesting alternative for a “high-tech” industry such as composite materials would be to review supply chain lessons and practices in “low-tech” industries such as food. The main motivation of this study is to identify lessons and practices that comprise innovations in the supply chain of a firm in a perceived “low-tech” industry that can be used to provide guidelines in the design of the supply chain of a “high-tech” industry, in this case composite materials. This work uses the case study/site visit with analogy methodology to collect data from a Spanish leading producer of fresh fruit juice which is sold in major European markets and makes use of a cold chain. The study highlights supply base management and visibility/traceability as two elements of the supply chain in a “low-tech” industry that can provide guidelines that can be used in the configuration of the supply chain of the composite materials industry.

## 1. Introduction

For many organizations all over the world, the development of new products and applications based on technological innovations and breakthroughs comprises a myriad of challenges. One industry which has been experiencing substantial growth because of new products and technological applications developed is the composite materials industry. At present the composite materials industry is growing steadily in many locations around the world as the expected global demand for carbon fibre will grow from 46,000 tonnes in 2011 to 140,000 tonnes by 2020 with production capacity being increased from 102,000 tonnes in 2011 to 129,000 tonnes in 2015, with the potential for further growth to 185,000 tonnes by 2020 [1]. In such context, inevitably many companies in the composites materials industry face the challenge of how to configure the supply chain to support the demand for resins and fibres, semifinished materials, components, and structures used in some of the most advanced and innovative solutions for the aerospace, automotive, construction, marine, oil and gas, rail, and renewables sectors. Composite materials can be classified as a “high-tech” industry given the substantial amount of financial resources spent on research and development (R&D) tasks. “High-tech” industries devote on average more than 10% of their expenditures to R&D [2] and conspicuous examples include aircraft and medical instruments.

Composite materials which encompass polymer matrix composites (PMCs), also known as fibre reinforced polymers (FRPs), consist of a matrix material, which is a polymer based resin, surrounding and supporting a reinforcement of some kind (typically fibres, particles, or flakes). The resultant PMC has properties that are advantageous compared to those of either the matrix or the reinforcement when used on their own [3].

The use of PMCs has resulted in the development of applications that are lighter, stronger, and stiffer and that can withstand corrosion among other properties. It is precisely these characteristics that have motivated companies in the automotive industry to increase their use of composite materials. According to German newspaper Handelsblatt, which cited sources inside the company, premium automaker BMW and carbon specialist SGL Carbon plan to double their joint production of carbon fibre to 6,000 tonnes a year. After the German business daily's report, the story has been also confirmed to Reuters by a person familiar with the situation—production in Germany and the US is planned for an expansion supported through more than 100 million euros investment, as BMW expects a strong demand for its i3 electric and i8 hybrid sports car [4].

In recent times it has been possible to witness multiple examples where innovations in composite materials are leading the way to state-of-the-art solutions in other sectors. The case of the Ford Fusion Lightweight Concept Vehicle illustrates this situation [5], as the replacement of metal-made components/parts by some made of composites/carbon fibre resulted in a mid-size sedan with the weight of a small compact car. Among the new components/parts used are carbon fibre seats, carbon fibre instrument panel, composites coil springs instead of solid steel, oil pan made of composites instead of cast aluminium, and carbon fibre wheels. However, Ford's prototype is not ready for high volume production and it is expensive to make. By introducing innovations in the design of the supply chain of this vehicle it may be possible to offset costs by consolidating parts [5]. In the aerospace industry there are multiple ongoing projects where fundamental structures such as fuselage and wings are made of composite materials instead of metals like aluminium. Bombardier's Learjet 85 perfectly illustrates the migration to composite materials [6, 7] requiring manufacturing processes which are different from those used in metal-based production that means having new suppliers and hence a new configuration of the supply chain.

The above cases in the automotive and aerospace industries illustrate that switching production of motor vehicles or aircraft to use components/parts made of composites materials represents a major challenge supply chainwise. The introduction of innovations in product and process requires having a supply chain capable of interacting and responding to opportunities on whether a technology can be or should be introduced depending on the availability of material, rate/process cascade effects, people and skills availability, and machine lead times among others.

There can be several paths that can be investigated to understand the configuration of the composites materials supply chain based on the introduction of innovations in product and process. Using the analogy methodology, one option is to look at developments in more mature industries, perhaps less advanced speaking for elements, lessons, and practices that can be used to understand the configuration of the composites supply chain. One “low-tech” industry that has introduced innovations in its supply chain and logistics operations is food. The food industry may be perceived as “low-tech”; however fresh food is perishable and requires temperature control; hence there is a need to bring supply chain and logistics innovations that can help to expand shelf-life and to make sure food reaches the hands of customers fresh and in optimum conditions. In the food industry the term “food chain” refers to the total supply process from agricultural production, harvest, or slaughter, through primary production and/or manufacturing, to storage and distribution, to retail sale or use in catering and by consumers [8].

Given the challenges facing the configuration of the composites materials supply chain the main motivation of this study is to identify elements that comprise innovations in the supply chain of a firm in a perceived “low-tech” industry. This work uses the analogy methodology to provide guidelines in the design of the supply chain of a “high-tech” industry. For that purpose the next section provides a review of examples of the work undertaken in some industries regarding supply chain configuration, a description of the typical supply chain structure characterizing the composite materials industry and the challenges affecting it, followed by a review of the literature regarding supply chain and logistics innovations in the food chain. This work uses the case study/site visit methodology to collect data from a Spanish leading producer of fresh fruit juice, one of the largest in Europe, which is sold in major European markets. The analysis of the case allows the identification of elements in the supply chain of the food chain that can be used to configure the composite materials supply chain. Conclusion and future opportunities are suggested at the end of the paper.

## 2. Supply Chain Structure and Challenges in the Composite Materials Industry

The traditional definition of supply chain management focuses on the creation of value by reaching beyond the traditional borders of a firm including suppliers, customers, and other stakeholders [9]. Furthermore, supply chain management integrates “key business processes from end user through original suppliers that provides products, services, and information that add value for customers and other stakeholders” [10].

The need for developing and implementing innovations in the supply chain comprising logistics operations is because of the positive effects on an organization, as it has been argued that logistics innovation can lead to positive operational performance by increasing efficiency, effectiveness, and service portfolios [11]. Furthermore, it has been discussed that logistics innovation is also positively related to the enhanced relationship with clients, growing sales, reputation, and financial performance [11].

The academic literature is limited regarding the supply chain of composite materials. Several studies can be found regarding supply chain configuration in industries such as semiconductor manufacturing [12, 13], vehicle assembly operations [14], textiles, and garment production [15] to mention just a few. Actually, some of these can be complex and difficult to manage. For example, in semiconductor manufacturing according to [12], there are many issues to be considered when setting up vendor-manufacturer relationships, including product price, product quality, company production, capacity, location, and transport costs. In summary, for semiconductor operations, an opportunistic supply chain design must be designed in response to a new microprocessor development [12]. On the other hand, not many studies can be found on composite materials and in particular the supply chain supporting this industry. Moreover, the study of the supply chain will continue to grow in importance as industries dependent on composites; for example, aerospace representing a global market of $4,471 bn continues to expand the adoption of structures and components made of composite materials. Other industries such as automotive has seen an increase in the use of composite materials as there is need for lightweight vehicle structures in order to meet new legislation regarding emissions reduction. Benefits of composite materials to automotive applications include reduced number of parts, reduction in tooling costs, good corrosion resistance, and excellent crashworthiness properties among others.

**Figure 1:**
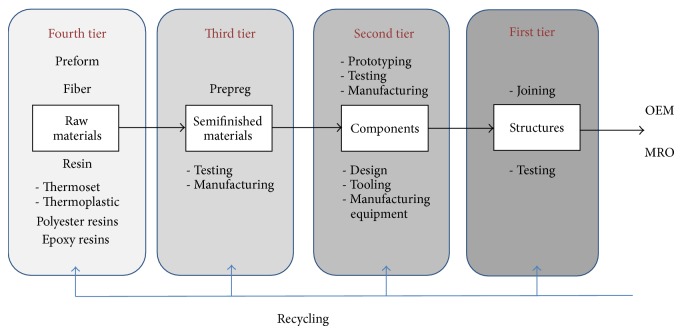
The composite materials supply chain.

Figure 1 shows a clear and simple structure of the supply chain of the composites materials industry. Although the location of a company can be well defined because of the type of product manufactured, still some organizations can play more than one role in the supply chain as some of them may be identified as a third-tier or a second-tier supplier depending on the type of product and industry they serve. Overall it can be highlighted that the industry shows low levels of vertical integration, similar to what has happened recently in semiconductors which evolved from a vertically integrated structure to a decentralized one.

In Figure 1, raw materials cover the supply of resins, fibres, and core materials. Common types of fibres include carbon fibre, carbon fibre precursors, and glass fibre. Resins include thermoset types such as epoxies, polyesters, vinyl esters, and phenolics. Examples of thermoplastics include PET and polycarbonates. Semifinished materials cover the materials that go through processes such as “prepreg” (preimpregnated composite fibres). Prepreg is a weave of fibres preimpregnated with a resin (i.e., epoxy resin). Common activities associated with semifinished materials include testing and manufacturing. Components refer to the development and manufacture of composite material parts. Key support activities at this stage include prototyping, testing, and manufacturing, not to mention the need for design, tooling, and manufacturing equipment. Structures refer to the manufacture of composite systems by joining several components. Hence, structures are commonly associated with joining components (i.e., wings of aircraft). At the end of the chain there are OEMs who manufacture end products that meet end customer/user requirements. Maintenance, Repair and Operations (MRO) organizations deal with spare parts and servicing.

Additionally, the use of recycled materials is a major operation that will impact the supply chain of the entire composite materials industry. Engagement in reuse activities has proven profitable in many industries but nowadays suppliers face increasing pressure to improve their environmental performance [16]. At the moment it is not entirely clear how the industry will embrace and implement the use of recycled materials. However, the composite materials industry must have in place clear provisions on how it will trace and recall materials and products. Hence, there is a big opportunity to expand the current knowledge base for the benefit of many in the industry.

The composite materials industry in the UK is mostly represented by SMEs with the majority playing roles of suppliers of resins and fibres, semifinished material, and components but not so much in terms of composite structures. At the same time most of the emphasis in their operations is on carrying tests and trials involving new manufacturing processes but little thought has been spent about the configuration of the supply chain they will need to support future increases in demand. Although simple in structure, the supply chain in the composite materials industry can give place to complex interacting relationships that navigate numerous industries. In the automotive industry, for example, some suppliers are working hard to bring down the costs of parts and components because carbon fibre is strong and lightweight. However, a company's ability to introduce a new technology may be severely constrained by several external factors.

In the particular case of the composite materials industry the introduction of a new technology may require additional people with skills and competencies that do not exist within the company itself, but which may also not exist within the general labor pool. This inevitably will affect the performance of the supply chain as the lead time for the introduction of the technology (based on facilities, equipment buildings, etc.) may be less than the time needed to recruit and train additional staff. Also technologies that favor increased production rates may be limited by constraints on material supply or the availability of the specific form of the materials required for the new process. Increased production rates may result in no benefit to a company if the rest of the supply chain cannot respond and undertake whatever additional actions are required at the same rate to complete the final product.

Manufacturing processes are having a major impact on the configuration of the composite materials supply chain. In fact innovation represented by the use of biomaterials including biofibres (such as soy hull, switchgrass, and their hybrids) for the fabrication of green composites has attracted major interest, as the degradation of this type of material does not emit toxic compounds in composting condition [17]. Hence, it is expected that the supply chain of composite materials will have to be reconfigured to cope with the use of biofibres but also in making changes to the reverse operation responsible for dealing with recycled materials.

## 3. Supply Chain Innovations in the Food Chain: Examining “Low-Tech” Industry for Answers to the Challenges Facing the Composite Materials Supply Chain

New product development and supply chain management and logistics research has clearly demonstrated that innovation is an important source of competitive advantage [18]. Hernández-Espallardo et al. [19] highlight that food is a mature industry where innovation activity is very important, with a strong emphasis on product innovations addressing new and differentiated demands as well as health, safety, and quality concerns, with market dynamics dominating the reasons for innovations. In recent years the food chain has adopted significant innovations as a result of managing complex supply chains involving food production, processing, distribution, and preparation and also as consequence of increasing customer awareness of food safety. Talking about innovations, the consolidation of the cold chain is perhaps one of the major innovation developments that have affected the food chain. Basically, the cold chain is a supply chain where temperature is under control; this means the product's temperature is under control until it reaches the hands of the customer. Consumer awareness of food safety and government food safety regulations are the major driving forces behind cold chain development [20]. It has been highlighted that in temperature sensitive and perishable products (TSPPs) logistics, a special type of supply chain management has been established, named cold chain management (CCM) [20]. Furthermore, it has been pointed out that customers expect that the products they order can be received safely, freshly, and on time as any temperature changes during the logistics process may cause loss of flavor or even spoilage [20].

One of the particular attributes of the logistics systems for chilled and frozen goods is the maintenance of product quality, which is dependent on the duration of delivery time and variation of temperature in the cold chain [21]. The physical logistics system of a cold chain is configured to minimize the cost of storage and transportation and to meet the requirements of product quality [20]. However, the cold chain requires additional capital investment in the forms of storage and transportation facilities, which ultimately reflects in higher operation costs.

The research questions arising from the paragraphs discussed above relate to the identification of elements comprising the supply chain of an organization in the food chain that can be used to configure the requirements of the composites materials supply chain. Research questions formulated include the following.

- What type of lessons on innovations in the logistics operations of the cold chain can be used in the configuration of the composites materials supply chain?
- What are the supplier base management and visibility/traceability policies in the food/cold chain that could be used in the “high-tech” composites materials supply chain?

## 4. Methodology

The case study methodology has elements that are useful for this research work. The methodology employed in case study research has been thoroughly explained by Yin [22] and generally the case study has some longitudinal dimension since it is conducted over a period of time. A ramification of the case study methodology used in this research is the site visit. Seaman [23] provides a detailed description of the use of site visits. According to her, a site visit is planned to obtain first-hand information from tours of specific facilities and services, interviews with individuals or groups, or observations of specific activities at the site. In addition, the site can be used to obtain reports, brochures, and examples of products or services made available at the site. Also site visits enable the opportunity to obtain first-hand information about users or activities in a particular setting. Another benefit of the site visit is the ability to evolve the data collection strategies on site, depending on the topics the evaluator determines are important to probe for obtaining additional information [23]. In particular this case pays attention to the identification of supply chain and logistics elements that could be used in the composite materials industry. The methodology emphasizes the identification and diagnosis of the challenges facing the management of the supply chain in the “high-tech” composite material industry. Also the methodology employed makes use of the analogy within the case investigated as it identifies transferable elements from the food industry supply chain to the composites materials supply chain. The analogy methodology has been widely used in case studies ranging from creating patents on new technologies [24] or used to determine the scale of R & D investments for renewable energy [25] and model predictive control strategies in supply chain management [26]. In the absence of enough data, guess-by-analogy has been considered prevalent and effective [27].

In order to have a better appreciation of the structure and operation of the composite materials supply chain, this research work relies on practitioner reports such as UK Composites 2013 [3]. This is fundamental to understand how the composite materials industry is affected by decisions in terms of responding to opportunities on whether a technology can be or should be introduced based on material availability and its associated effects as well as the use of recycled materials by different tiers of suppliers.

Given some of the aspects highlighted in the above paragraphs, the analysis of the supply chain of composite materials requires a high level of visibility for the purpose of tracking and tracing materials. For that reason the concept of supply chain product visibility is applicable to some extent to the needs of the composite materials industry, in particular the conditions observed in the UK. This concept involves developing and keeping a record of the product's materials and components, its physical state throughout the supply chain, the product's forward movement to the user-customer, customer's experience of the product, and the reverse logistics and reuse or termination of the product [28].

## 5. Case Analysis: Results and Discussion

### 5.1. Details of the Participating Organization in the Food Chain

The organization participating in this case is identified as “*Company A.*” Due to confidentiality issues the identity of the company cannot be disclosed.* Company A* is one of the largest food groups in Spain and Western Europe and it is dedicated to the commercialization of fresh fruit (mainly citrus and grapes), flowers, ice-cream, and the production of fruit juice including chilled and long life presentations. The latter represents its most important line of business. The company is the fifth largest group dedicated to the production of fruit juice in the world and its headquarters and largest production site are based in South Eastern Spain.* Company A* has about a network of 500 suppliers spread all over the world and 70% of its production is exported from Spain to markets worldwide.

In terms of product variety the company has been able to develop competitive economies of scale and economies of scope with a current range of products in the order of 900 SKUs. Any new product to be developed requires about a year of development. Final product innovation is fundamental for the long term success of this business. Hence, the company recognizes that the ultimate goal is to bring to market new varieties with characteristics that create real additional benefit to the consumer. The company has joined two structure programs for the development of new products. One is Special New Fruits Licensing Ltd. (http://www.snfl.eu/) which is a structure exclusively dedicated to bringing together the best specialists in hybridization with the best producers in the world and which include advantages such as

- the use of early or late fruit,
- the use of special varieties or niches,
- wide range,
- superb quality,
- new flavors,
- unbeatable features for transport and storage.

**Figure 2:**
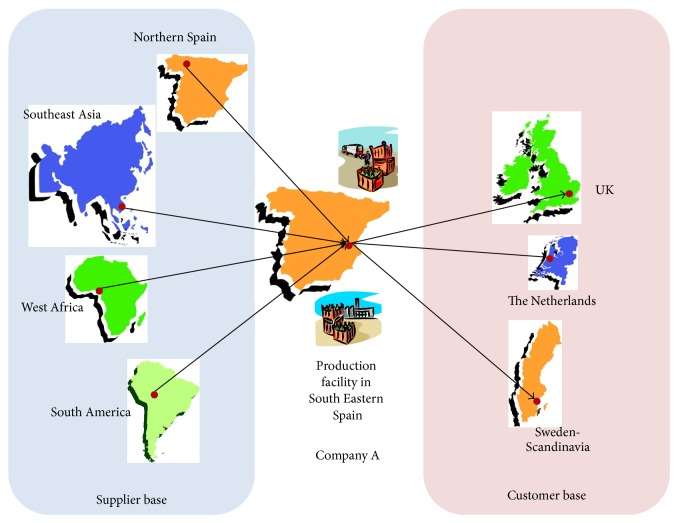
*Company A* supplier and customer base extends around the world.

A simplified representation of the supply and customer base for* Company A* is depicted in Figure 2.

The company is also member of Citrus Genesis (http://www.citrusgenesis.com/), a working structure oriented towards scientific research and which works on the genetic heritage of various citrus varieties in order to learn about the product and its potential for cultivation and also meet consumer expectations.

### 5.2. Supply Chain and Logistics Operations and Innovations

**Figure 3:**
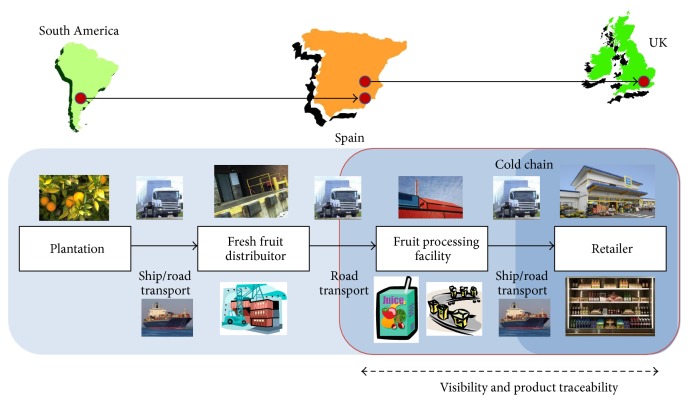
Supply chain with fruit suppliers in South America and final market in the UK.

Because chilled fruit juice is the most important line of business, the company makes extensive use of cold chain for shipping finished products to customers in European markets. Its largest customer base is located in Northern Europe and that includes the United Kingdom, The Netherlands, Germany, and Scandinavian countries.* Company A* ships 200 million liters of fruit juice every year and it uses 10,000 truck trips. The lead time associated with the delivery of customer orders within Europe is from three to five days. For example, it takes three days for a shipment to reach the UK and shipments to Scandinavian countries usually take five days. The UK represents the group's single largest market in Europe for chilled fruit juice. Main customers in the UK include major supermarket chains such as Waitrose, Marks & Spencer, and Tesco.  Figure 3 depicts a representation of the supply chain in place for* Company A's* production of chilled fruit juice delivered to the UK with fruit sourced from South America.

In Figure 3, the dark segment of the chain with a red ring means that the cold chain runs between the fruit processing facility producing the chilled fruit juice and the retailer facilities where the product is sold to the end consumer. At the same time, the development of the cold chain motivated the development of visibility and product traceability capabilities. Between the orchards/plantations, the fresh fruit distributor, and the fruit processing facility usually there is no cold in chain involved, unless the fruit sourced comes from the southern hemisphere.  Figure 3 also shows a situation in which fruit suppliers may be located out of Spain, in South America; hence the produce has to be shipped to Spain; on arrival in the port the fruit has to be transported to the processing plant and once the product has been produced it is sent by ship and road to markets in Northern Europe, in this case the UK.

The company has been implementing transport policies that are helping it to reduce its CO_2_ footprint at a time its most important customers like large supermarket chains are demanding their suppliers to meet designated sustainability and carbon reduction targets. For that reason* Company A* has adopted short-sea shipping transportation to deliver its products to the UK market and because of the implementation of this solution the company has received an award for innovation from UK-based Waitrose supermarkets. The way this solution works is that the truck leaves the production plant and travels by road to the port of Bilbao in Northern Spain; from there it travels on a ferry ship to the port of Bristol or the port of Liverpool. On arrival the truck travels to inland retailer facilities including stores and distribution centers.

*Company A* has experienced in recent years significant fluctuations in the availability of transport. For example, the company is always in negotiations with shipping companies about pricing, as the months from May to September are when demand for chilled fruit juice increases during the year.* Company A* relies mainly on shipping and road haulage providers as rail is not a suitable option. Indeed, the company agrees that rail does not have the flexibility required by their business. It can be added that retailers of chilled fruit juice have various needs and different delivery requirements. For this reason* Company A* has worked closely with them to improve vehicle delivery utilization by ensuring full truck-loads, reduce the number of deliveries per week, eliminate late deliveries, and guarantee a 100% service level.

The supply chain of the organization in the food/cold chain has two representative elements that need further discussion: supplier base management and visibility/traceability.

#### 5.2.1. Supplier Base Management: Augmentation Not Reduction

Opposite to what prevails in other industries such as automotive in terms of supplier base reduction, the fruit juice manufacturing industry and in particular* Company A* has a policy of keeping a large number of suppliers rather than looking to reduce its pool base. Actually, a reduced supplier base is seen as something negative. Fruit can be very diverse; hence there is a constant need to have suppliers in various countries which can specialize in certain types of fruit (e.g., pineapple from Costa Rica or apple varieties such as pink lady from Italy, Austria, and South Germany). Also* Company A* has in place contingency plans which facilitate replacing a supplier in case of emergency. The situation regarding supplier base management could be labelled as supplier augmentation rather than supplier rationalization. On the other hand the number of customers has not been rationalized either, as* Company A* serves over 40 supermarket retailer chains across Europe. Indeed, retail clients sell their own brand; hence,* Company A* can achieve economies of scope.

#### 5.2.2. Visibility and Traceability

The safety of the consumer is paramount; hence stringent quality and biological controls are present in the production of chilled fruit juice. For this reason* Company A* has developed an effective system capable of tracing batches of product in case of problem using barcode technology. In case of an incident detected after leaving the factory in Spain, a truck can be ordered to stop in France and leave the pallet that needs to be recalled. In order to facilitate traceability the company has embraced inventory policies where it holds between 1 and 1.5 weeks of buffer stock which insures 100% customer service. This reduces significantly the need to trace large inventory levels.

### 5.3. Discussing Supply and Logistics Innovations from a Perceived “Low-Tech” Industry That Can Be Used in “High-Tech” Industry

In the case of* Company A*, having an extensive supplier network that guarantees access to raw materials is fundamental for running the business and to ensure delivery of customer orders. Similarly, the emerging supply chain for composites materials requires a significant level of robustness where suppliers comprise multiple value chains linked together and where the introduction of change, such as a new manufacturing technology or a new requirement by a customer, at any one place will result in logistical impacts and constraints across the supply chain.

**Figure 4:**
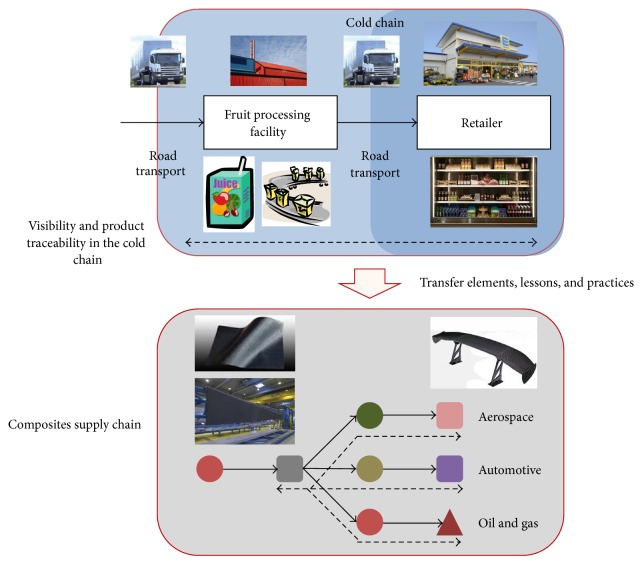
Cold chain elements transferred to the composites supply chain.

The use of the analogy methodology can make possible to identify segments within the examined supply chain in the food industry that possess the level of control (visibility and traceability) that is required in composites materials. In some cases the operation of the composites materials supply chain is subject to stringent control and tolerances required by other sectors it serves such as aerospace and oil and gas. Hence, the cold chain used by* Company A* can teach valuable lessons that can be used in the visibility and traceability configuration of the composites materials supply chain. Indeed, the control of parameters is perhaps one of the most important ones as it has been explained that temperature control is required during the logistics process that follow production to customer delivery as changes in the temperature may cause loss of flavor or even spoilage of the final product before it even reaches the hand of the end consumer. Also, the need for visibility and traceability makes possible to stop a shipment from reaching the customer in situations where variation in the temperature has been detected during the delivery process. Similarly, a high degree parameter control is needed in the composites materials supply chain as some source parts/components have to meet stringent specifications and tolerance levels; hence immediate visibility and traceability that allow a quick recovery of a product as well as recycling have become essential characteristics that must be present in the composites materials supply chain.  Figure 4 shows the transferability of the supply base and visibility/traceability elements used in the cold chain, where visibility and traceability are in dotted lines and can also be used in the composite materials supply chain.

In the supply chain illustrated in Figure 4, composites-sourced industries, aerospace, automotive, and oil and gas supply chains are transferred visibility and traceability lessons and practices as indicated in the dotted lines. Elements of visibility and product traceability are required as new materials, processes, and products are developed, making them even more important as the composites supply chain feeds other “high-tech” industries.

The composite materials industry is a key supplier to other “high-tech” industries such as aerospace, automotive, construction, marine, oil and gas, rail, and renewables among others. However, in recent years the tendency in some “high-tech” industries like automotive has been to significantly reduce the size of their supplier base. Indeed, in recent years automotive OEMs and large first-tier suppliers have adopted supplier base reduction programs but it seems that the composites materials supply chain may not be susceptible to supplier base reduction, as the number of companies with the technological know-how, materials availability and sourcing, people, and technical skills is already in short supply. Most likely, an approach similar to the one adopted by* Company A*, which relies on keeping a broader supplier base is the most appropriate supply base management policy for the future as it has the potential to support contingency plans and promote specialization among the supplier base. As the supply chain of chilled fruit juice has a multinational supply base, interestingly enough the supply chain of composite materials can be part of a large multinational supply base for “high-tech” industries such as aerospace. For example, the composite wings of Bombardier's new C series planes are made in Northern Ireland, from there the wings are shipped to Mirabel, Quebec, Canada, for final assembly.

## 6. Conclusions

Using a case study based on the analogy methodology, this paper presented a study about how an organization in a perceived “low-tech” industry has embraced supply chain and logistics innovations that can provide invaluable elements, lessons, and practices that can be used to provide guidelines to help configure the structure and processes of “high-tech” industries, in particular the development of effective supply chain management configurations. The food chain involving the production and commercialization of chilled fruit juice and which makes extensive use of the cold chain has two elements, visibility and traceability, that can be transferred to emerging “high-tech” industries such as composite materials.

Just as in the food chain consumer awareness of food safety and government food safety regulations have become the major driving forces behind innovations such as the cold chain while enabling visibility and track and trace capabilities, we can expect that in the future customer requirements, government agencies and legislation will exert a substantial level of influence that will determine traceability and recovery actions taking place in the composite materials supply chain. Industries such as aerospace and medical devices/equipment will be immediately affected by government legislation because of the safety element attached to the products they make.

This work has the opportunity to make a significant contribution by expanding the existing knowledge about the supply chain of composite materials. Overall, the research has several practical implications given the forecasted growth of the composites materials industry on a global basis and at a time some countries are developing capabilities to become global hubs for composites materials.

Finally, the food chain/cold chain has clearly challenged current trends adopted in some “high-tech” industries like automotive involving supplier base rationalization. The composites materials industry is relatively new and modest in size, comprising suppliers which are small and medium enterprises with different levels of technological expertise, capacity, equipment, rate/process, people, and skills, among others. It seems that reducing the number of suppliers will not help as the composites materials industry has not reached maturity.
